# Clinical audit effectively bridges the evidence-practice gap in chronic subdural haematoma management

**DOI:** 10.1007/s00701-016-3063-2

**Published:** 2017-01-11

**Authors:** Jignesh Tailor, D. Fernando, Z. Sidhu, R. Foley, K. D. Abeysinghe, D. C. Walsh

**Affiliations:** 1 Department of Neurosurgery, King’s College Hospital, Denmark Hill, London, SE5 UK; 2 King’s College London School of Medicine, Strand, London, WC2R UK; 3 Institute of Psychiatry, Psychology & Neuroscience, King’s College London, London, SE5 UK

**Keywords:** Chronic subdural haematoma, Burr-hole evacuation, Subdural drain, Recurrence

## Abstract

**Background:**

Placement of a subdural drain after drainage of chronic subdural haematoma (CSDH) has been shown to reduce the rate of recurrence in several randomised controlled trials (RCT). The most recently published RCT was from Cambridge, UK, in 2009. Despite class I evidence for the use of subdural drains, it is unclear whether these results have been translated into clinical practice. In this clinical audit we review the use of subdural drains in our institution before and after the publication of the 2009 RCT results.

**Methods:**

A longitudinal retrospective study was performed on all adults having burr holes for CSDH between January 2009 and January 2014. Case notes were analysed to determine subdural drain use, re-operation for CSDH recurrence and post-operative complications. The audit loop was closed with data collected from August 2015 to January 2016.

**Results:**

Thirty-one per cent of patients had subdural drains placed at operation. Drain placement was associated with lower reoperation rates (8% vs. 17%, p = 0.021) without increasing complication rates. Drain usage doubled after publication of the Santarius et al. (2009) trial but we observed persisting and significant variability in drain utilisation by supervising consultants. The use of drains in the department increased from 35% to 75% of all cases after presentation of these results.

**Conclusions:**

The use of subdural drains in our unit reduced recurrence rates following drainage of CSDH and reproduced the results of a 2009 clinical trial. Although the use of subdural drains doubled in the post-trial epoch, significant variability remains in practice. Clinical audit provided an effective tool necessary to drive the implementation of subdural drain placement in our unit.

## Introduction

Burr-hole evacuation of chronic subdural haematoma (CSDH) is a common neurosurgical operation worldwide, and the use of a subdural drain following haematoma evacuation has been shown to reduce the risk of re-operation [2, 6, 7, 11, 13, 14]. In 2008, a survey of British neurosurgeons highlighted that insertion of a subdural drain was not common practice in the UK [12]. A year later, Santarius et al. published the results of a randomised controlled trial demonstrating a reduction in re-operation rates from 24% to 9% when a subdural drain could be placed safely intra-operatively [11]. These results were similar to those found in previously reported trials [7], providing class I evidence for the use of subdural drains following burr-hole evacuation of clots. The impact of these clinical trials on clinical practice has not been studied. The aim of this study was to compare the rates of subdural drain usage at our institution in the post-trial epoch (2010 onwards) with that before the publication of Santarius et al. to examine the impact of the trial results on clinical practice.

## Methods

We performed a retrospective study of subdural drain usage in all consecutive patients receiving burr-hole drainage for CSDH at King’s College Hospital between January 2009 and January 2014. Patients were treated by clinical teams, and each team was directed by a Consultant Neurosurgeon who defined the standard operative procedure for the team. Patients who underwent burr-hole drainage between January 2010 and January 2014 were stratified into the ‘post-trial’ epoch (3 months after the Santarius et al. trial results were published [11]). Paediatric patients and patients with CSF diversions in situ were excluded. Data on patient demographics, subdural drain use and surgical complications were collected from the clinical notes. The primary outcome measure was re-operation for recurrent chronic subdural haematoma within 6 months. Statistical analysis was performed using SPSS v20 with chi-squared values derived from four-fold tables for binary data. A *p* value of < 0.05 was considered significant. Graph Pad Prism v7.0 for Mac OSX, GraphPad Software, La Jolla CA, USA, www.graphpad.com, was used for other calculations and in generating figures. To close the audit loop, these data were also collected retrospectively for all consecutive patients receiving burr-hole drainage for CSDH between August 2015 and January 2016 (6 months following presentation of the primary results).

## Results

Four hundred twelve burr-hole drainage procedures were performed for CSDH between January 2009 and January 2014. Seventeen patients were excluded from the study in total (because of incomplete data, presence of a shunt or placement of a sub-galeal drain over the burr hole). Of the remaining 395 patients, 123 (31.1%) had a subdural drain inserted (Table 1).

**Table 1:** A comparison of re-operation rates and patient characteristics between the ‘drain’ versus ‘no drain’ groups

	Total	Drain	No drain	*P* value
No. of procedures (%)	395	123 (31.1%)	272 (68.8%)	
No. of re-operations (%)	56 (14.1%)	10 (8.1%)	46 (16.9%)	0.021*
Mean age	73.1	75.6	71.9	
Average GCS	14	14	14	
Male (%)	296 (74.9%)	88 (71.5%)	208 (76.5%)	0.296
2 Burr holes (%)	237 (60.0%)	77 (62.6%)	160 (58.8%)	0.477
Bilateral CSDH (%)	76 (19.2%)	26 (21.1%)	50 (18.4%)	0.519
Dexamethasone used (%)	13 (3.3%)	3 (2.4%)	10 (3.7%)	0.523
Anticoagulants (%)	98 (24.8%)	30 (24.3%)	68 (25.0%)	0.897

*Statistical significance (P < 0.05)

**Fig. 1:**
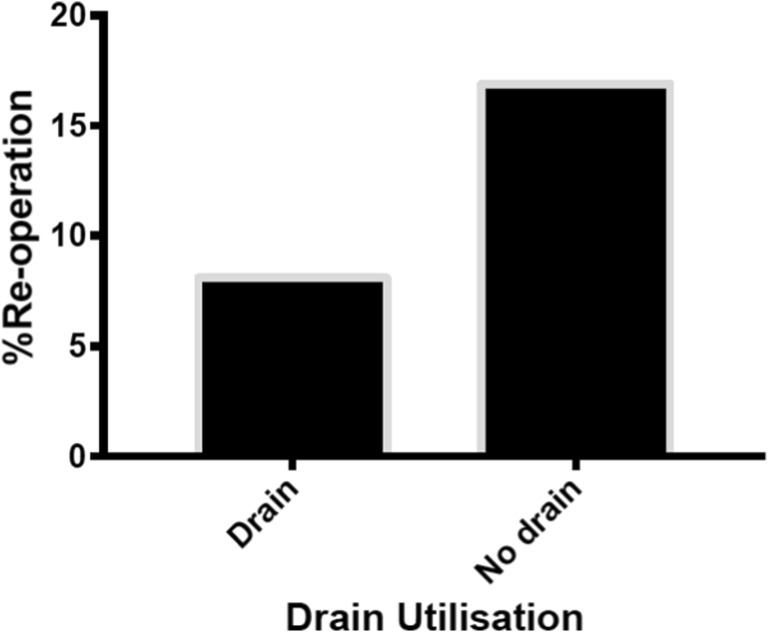
Re-operation rate with the use of drain is significantly lower compared to patients who received no drain (*P* < 0.05)

The re-operation rate was significantly lower in the patients who had a subdural drain inserted (8.1%) compared to those that had no drain (16.9%) (*P = 0.021*) (Table 1 and Fig. 1). The two groups were very similar with respect to age, gender, Glasgow coma score (GCS) at presentation, % that had bilateral CSDH as well as the percentage of patients who were on anticoagulants at presentation (Table 1). A minority of patients (3.3%, n = 13) were prescribed dexamethasone peri-operatively. Three patients receiving steroid treatment received subdural drains and ten did not. There was no significant difference in the overall rate of post-operative complications following subdural drain insertion compared to no drain. The rates of infection, seizures and pneumocephalus were similar (Table 2).

**Table 2:** A comparison of complication rates for patients who received a subdural drain versus no drain (percentage of the total number in each group is given in brackets)

	Total	Drain	No drain	*P* value
Number of procedures	395	123	272	
Pneumocephalus (%)	25 (6.3)	10 (8.1)	15 (5.5)	0.322
Seizure (%)	22 (5.6)	8 (6.5)	14 (5.1)	0.586
Infection (%)	6 (1.5)	2 (1.6)	4 (1.5)	0.907

**Fig. 2:**
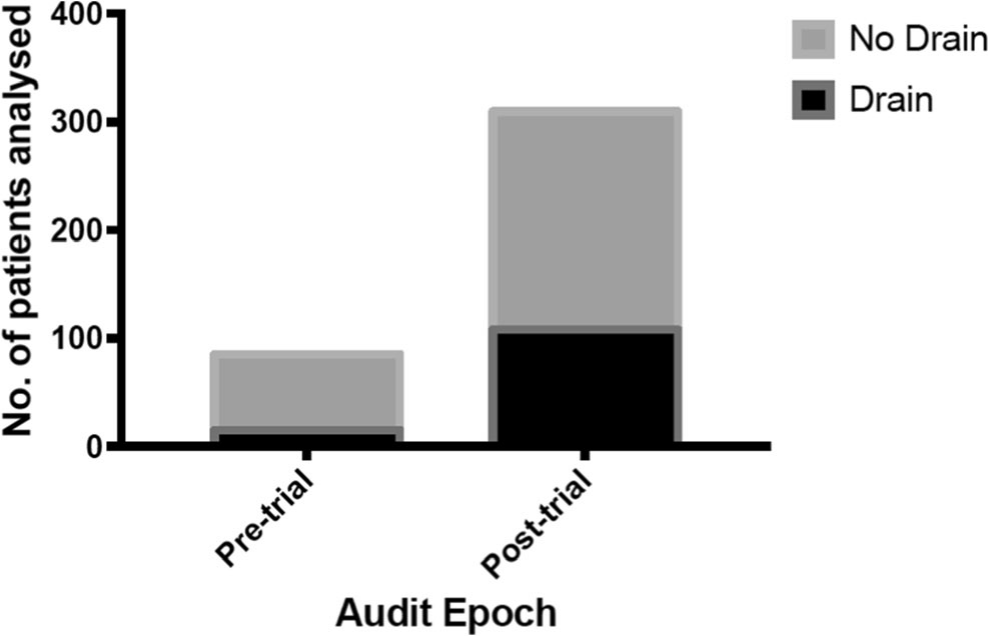
The use of subdural drains in the pre- and post-trial epochs

Next, we compared the subdural drain usage before and after the publication of the Cambridge RCT (Santarius et al., 2009). We observed that the use of drains increased from 17% (15/90) to 35% (108/310) in the post-trial epoch (Fig. 2). Given that the use of drains is supported by class I evidence this was still much lower than anticipated. No significant difference in the overall re-operation rates between the pre-trial (9.4%) and post-trial epochs (15.5%) was observed (P = 0.15).

**Fig. 3:**
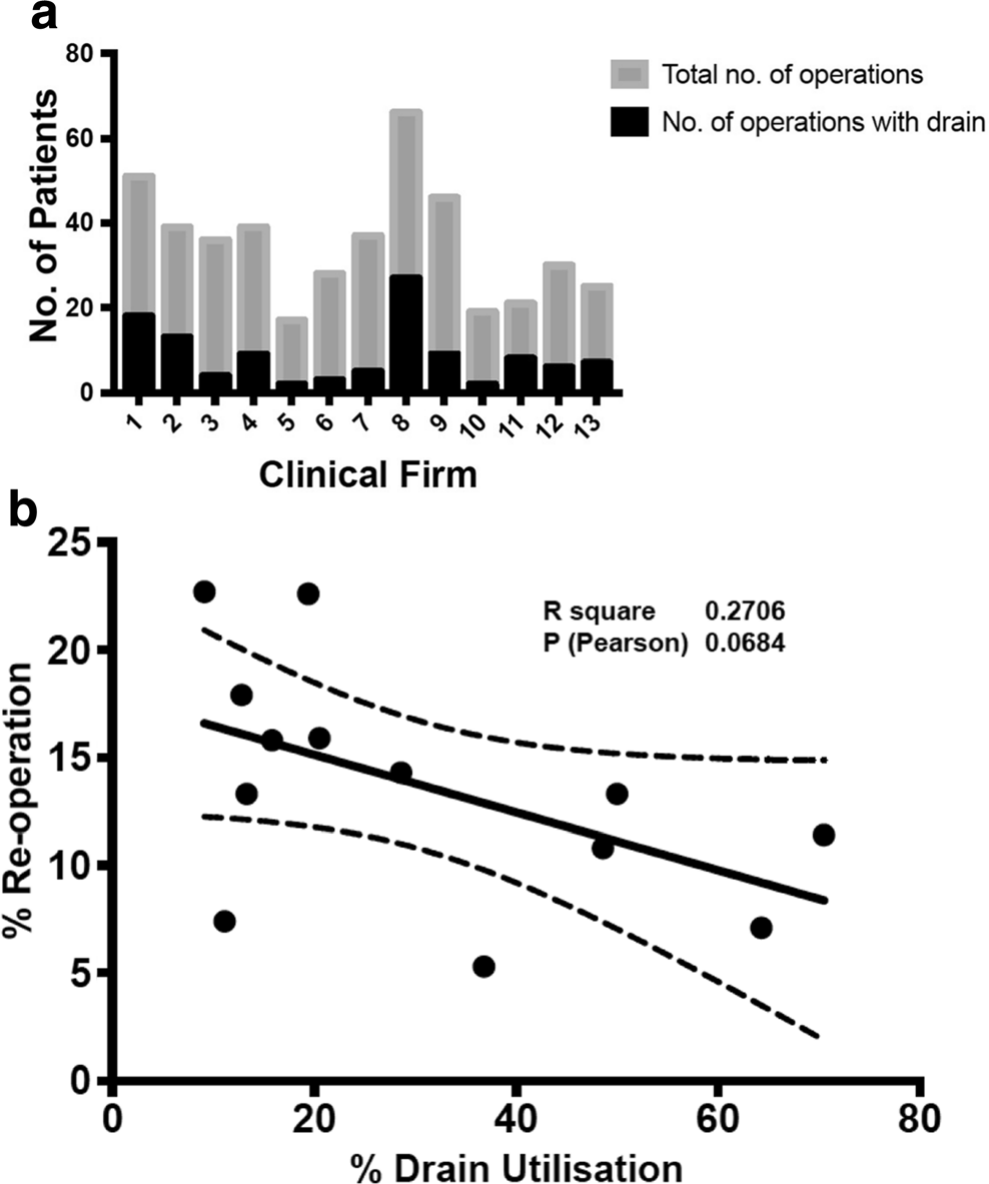
The variability in clinical practice amongst consultants. **a** The absolute numbers of operations with subdural drain in relation with the total number of burr-hole operations performed for CSDH. **b** The re-operation rates are plotted against subdural drain usage for each consultant. There is a trend towards lower re-operation amongst consultants with higher subdural drain usage rates (R^2^ = 0.27191)

To further investigate this, we looked at the operations performed under the care of individual consultants within our department. We found a large variability in drain usage between consultants (Fig. 3a). Whilst some consultants specified subdural drains in 70% of cases, there were others who specified subdural drains in only 10% of cases. The majority of consultants had inserted subdural drains in less than 50% of their cases. Numbers with these groups were small and although a trend towards lower re-operation rates may be suggested in clinical teams with a higher rate of subdural drain usage, the difference was not statistically significant (Fig. 3b) (R^2^ = 0.270, Pearson 0.0684).

**Fig. 4:**
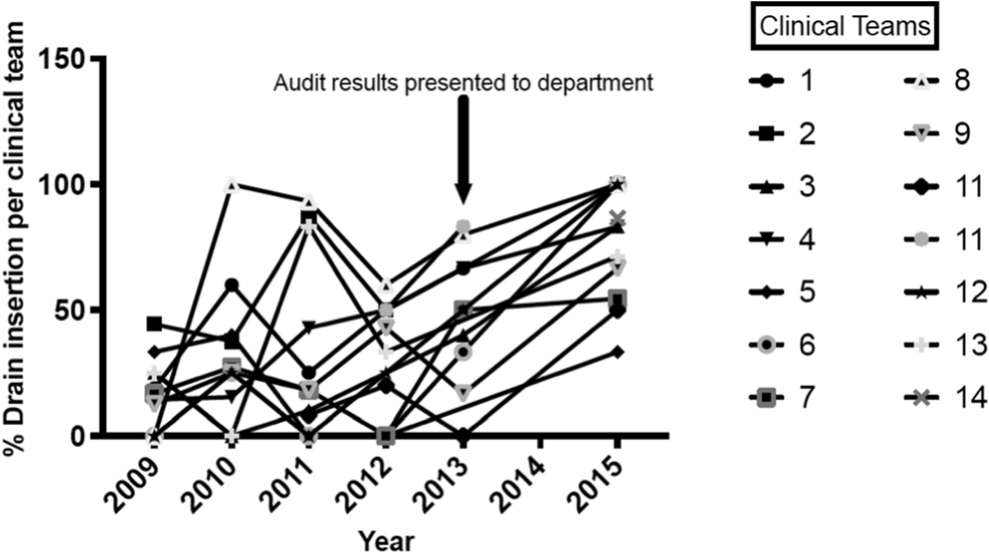
The percentage drain insertion per clinical team over the period of 2009 to 2016. The % drain usage in each clinical team is plotted per year. Data points on the graph were omitted if the clinical team performed fewer than three CSDH drainage procedures in that year. Only one clinical team (team 8) convincingly adopted the practice of using subdural drain in 2010, following the publication of the Santarius et al. trial in September 2009. The practice was variable amongst the other clinical teams between 2010–2013. Following the audit of drain usage from January 2009 to January 2014, and subsequent presentation to the Morbidity and Mortality meeting in Spring 2015, there was a notable increase in % drain use by the majority of the clinical firms in 2015

The results were presented to the department Morbidity and Mortality meeting in Spring 2015. Subsequently a re-audit of subdural drain usage and re-operation rates was performed for all CSDH operations between August 2015 and January 2016. Seventy-three operations were performed during this period, and no exclusions were made. Of these 73 patients, 55 received a subdural drain (75%). This was a notable increase compared to the subdural drain usage in the post-trial epoch (35% between 2010–2014). Re-operation for CSDH recurrence was performed in three patients who received a subdural drain (5.5% of all patients receiving a subdural drain) and three patients who did not receive a subdural drain (16.7% of all patients with no drain). The overall re-operation rate for the department in this 6-month re-audit period was 8.2%. We then performed an analysis of subdural drain use per clinical team from 2009 to 2015 (Fig. 4). This showed that only one clinical team convincingly adopted the practice of using a subdural drain in 2010, following the publication of the Santarius et al. trial in September 2009. The practice was variable amongst the other clinical teams from 2010–2014. Following the presentation of the audit results to the department in 2014, there was a notable increase in % drain use by the majority of the clinical teams.

## Discussion

In this study, we retrospectively reviewed the use of subdural drains in a single centre before and after publication of the 2009 RCT [11]. We found that the use of a subdural drain following evacuation of CSDH in the post-trial epoch increased, but there was significant variability in practice and the majority of consultants were still specifying subdural drains in less than 50% of cases. These results demonstrate that, although supported by class I evidence, the placement of subdural drains had not yet been embedded in clinical practice.

There are a number of factors that may have contributed to these results. First, whilst the supervising consultant is responsible for operations, burr-hole drainage of CSDH is often performed by junior neurosurgeons in the department who may have a higher threshold for placing a subdural drain in the emergency setting and out of hours. However, in our institution, senior clinical fellows supervise the junior registrars out of hours, and it is standard practice to discuss the operative strategy with the supervising consultant peri-operatively. In addition, only a small proportion of patients was excluded in the Cambridge trial because insertion of a subdural drain was judged ‘unsafe’ (28/269) [11]. Therefore, lack of experience and/or technical expertise is unlikely to be the sole explanation for the low rates of subdural drain placement in our initial cohort.

Variability in clinical practice is often driven by personal experiences of the operating surgeon. Many surgeons have anecdotally refrained from using subdural drains because of previous complications [1]. Our study and previous clinical trials demonstrate that the placement of subdural drains does not significantly increase complication rates [7, 11]. The organisation of the clinical service may also play a role. In the structure where this study was carried out, autonomous clinical teams supervised by a Consultant Neurosurgeon deliver care. Some of these teams have standardised operating procedures specifying the insertion of a drain, while others do not.

It is well recognised that the passive dissemination of information, in the absence of exploring behavioural barriers and facilitators to practice change, is ineffective [3, 10]. Knowledge translation is defined as ‘the science of developing strategies to integrate evidence-base knowledge into health policy and practice, based upon the understanding of behavioural drivers of clinical practice’ [4]. Our study demonstrates that the publication of class I evidence for subdural drains in CSDH in 2009 was insufficient on its own to alter practice and that clinical audit and local feedback to clinical teams were a necessary requirement to embed this change.

Although it is not possible to comment on whether similar evidence-practice gaps exist in other neurosurgery units within the UK or Europe, the failure to translate research into clinical practice and policy is a recurring and consistent finding in health services research [4]. McGlynn and colleagues observed that patients in the USA received 55% of recommended care and that quality varied by medical condition ranging from 79% of recommended care for senile cataracts to 11% of recommended care for alcohol dependence [8]. Similar findings have been reported globally in both primary care and specialty-provided care and in care provided by all disciplines [5]. Our study specifically illustrates the ‘evidence-practice gap’ in the use of subdural drains following burr-hole drainage of chronic subdural haematoma. Our results confirm that local interventions such as educational meetings, journal clubs, clinical audit and practice guidelines are necessary strategies to ensure that health care professionals provide evidence-based care for patients.

The National Institute for Health and Care Excellence (NICE) publish evidence-led practice guidelines in the UK. NICE have published guidelines on acute head injuries [9], but there are no specific guidelines on the management of chronic subdural haematoma. Burr-hole drainage of the chronic subdural haematoma is performed relatively frequently and with an aging population. It is anticipated that the surgical workload related to chronic subdural haematoma will increase. Publication of appropriate evidence-based guidelines may help to accelerate the translation of clinical trial results into clinical practice.

Of course, our study has inherent limitations. It is a retrospective study in a single, albeit busy, neurosurgical centre. The placement of drains was non-randomised, so we cannot exclude the possibility of selection bias in the results. Nevertheless the study was designed to be an audit of clinical practice against a well-published evidence base. The re-audit results showed a marked improvement in subdural drain usage following the presentation of the initial results to the department. Our study therefore demonstrates the power of clinical audit as a tool to implement clinical practice and to confirm the safety and efficacy of operative techniques.

## Conclusions

The conduct of this audit has shone a light on an unanticipated variation in clinical practice. The use of subdural drains in our unit reduced recurrence and reproduced the results of a 2009 clinical trial, significantly reducing the rate of re-operation. Subdural drain use is gradually increasing but there has not been a wholesale adoption in practice as yet. Clinical audit has provided a tool necessary to drive the implementation of this practice in our unit as well as to confirm its safety and efficacy.
